# Impact of Malocclusion on the Quality of Life of Brazilian Adolescents: A Population-Based Study

**DOI:** 10.1371/journal.pone.0162715

**Published:** 2016-09-30

**Authors:** Luciana Freitas Gomes e Silva, Erika Bárbara Abreu Fonseca Thomaz, Heloiza Viana Freitas, Alex Luiz Pozzobon Pereira, Cecília Cláudia Costa Ribeiro, Cláudia Maria Coelho Alves

**Affiliations:** 1 Graduate Program in Dentistry, CEUMA University, São Luís, Maranhão, Brazil; 2 Graduate Programs in Dentistry and Public Health, Federal University of Maranhão, São Luís, Maranhão, Brazil; 3 Graduate Program in Dentistry, Federal University of Maranhão, São Luís, Maranhão, Brazil; University of Washington, UNITED STATES

## Abstract

The objective of this study was to evaluate the impact of malocclusion on the quality of life (QOL) of adolescents in Brazil. We carried out a cross-sectional study in a sample population of 1015 schoolchildren aged 12 to 15 years from São Luís, Maranhão, Brazil. The explanatory variable was malocclusion, evaluated on the basis of the normative need or the adolescent’s self-perceived need for dental treatment. Normative need for dental treatment was determined by professional diagnosis, made on the basis of Angle’s classification, the Dental Aesthetic Index, and other morphological deviations (e.g., posterior crossbite, posterior open bite, and deep overbite). We analyzed the impact of malocclusion on the QOL using the Portuguese version of the Oral Health Impact Profile-14. Associations were estimated by using the prevalence ratio (PR) in Poisson regression analysis, with hierarchized modeling. An alpha of 5% was adopted as the criterion for statistical significance. The QOL of adolescents was impacted by malocclusion, classified by a normative need for treatment according to the Dental Aesthetic Index (PR = 1.27; 95% confidence interval [CI] = 1.03–1.56) or by the self-perceived need for treatment (PR = 2.54; 95% CI = 1.81–3.56). Certain sociodemographic variables, including the head of the family (PR = 1.52; 95% CI = 1.02–2.23), greater educational level of the head of the family (PR = 0.32; 95% CI = 0.17–0.61), and female sex (PR = 1.40; 95% CI = 1.05–1.89), had negative associations with QOL. We conclude that malocclusion has a negative impact on the QOL of adolescents, associated with socioeconomic conditions and the cosmetic effects of malocclusion.

## Introduction

The concept of oral health-related quality of life (QOL) refers to the impact that oral health or disease has on a person’s day-to-day activities and general wellbeing [1]. Oral diseases and disorders can have negative effects on the lives of those who have them [2]. For example, facial appearance affects how a person perceives themselves and is perceived by society [3]. Therefore, QOL is a dynamic construction [4]. Each person has their own self-perceptions, which are influenced by their way of life, past experiences, hopes for the future, dreams, and ambitions [5].

There has been increasing interest in QOL as it relates to the oral health of adolescents, whose lives are likely to be negatively impacted by oral disorders [6]. Various studies have been conducted to analyze the impact of malocclusion on adolescents’ QOL and have found that malocclusion is associated with higher levels of dissatisfaction with appearance, and have the potential to negatively impact QOL [3,7–9]. However, socioeconomic status, the home environment, and familial influences play key roles in determining an individual’s oral health. Thus, in addition to functional dimensions, the psychosocial dimensions of oral health must be considered when seeking to implement and evaluate oral health interventions [10].

One instrument that is commonly used to evaluate the impact of oral health problems on QOL is the Oral Health Impact Profile (OHIP)-14, which is a shortened form of the original OHIP-49 developed by Slade [11]. The OHIP-14 has been validated for Brazilian population[12]. This instrument was originally developed through studies of elderly patients, but has shown good success when used to evaluate the impact of oral problems on the QOL of adolescents. The OHIP-14 has good psychometric properties, which are similar to those of the original instrument.

To evaluate the impact of malocclusion on QOL, we need to consider the different domains that can be affected, as well as their relationship with personality traits and psychosocial or socioeconomic factors. Some people with serious malocclusion are satisfied with or indifferent to the appearance of their teeth, while others are worried with minor irregularities [7,13]. Thus, the objective of this study was to evaluate the impact of malocclusion and the perception of the need for orthodontic treatment on the QOL of adolescents, also considering socioeconomic factors.

## Methodology

This research was approved by the Research Ethics Committee of the University Hospital of the Federal University of Maranhão (protocol n° 2429/2010-10). Written informed consent was obtained from the State Department for Education, the directors of the participating schools, and the parents/guardians of the schoolchildren.

We carried out a cross-sectional study in a population of adolescent boys and girls (age range: 12–15 years) who were enrolled in primary education schools in São Luís, MA, Brazil. Eligible participants were students who were regularly enrolled in years 5 to 9 of primary education, were aged 12 to 15 full years at the moment of the exam, and had not previously received any type of orthodontic treatment. The exclusion criteria were: adolescents with mental disorders, based on parents and teachers report (n = 0); who didn´t have first permanent molars in the dental arcade (n = 20) based on clinical examination; who refused to participate in the study (n = 07); or who were not present in school at either of two evaluation visits (n = 11).

The sample size was calculated by using the Epi Info software package, version 6.0 [14]. We estimated that a sample of 336 pupils would have a power of 90% and a confidence level of 95% to identify significant prevalence ratios (PR) exceeding 1.6. We assumed that the prevalence of impact on QOL among unexposed adolescents (no malocclusion) was 29.57% [15], and that there was a 1:1 proportion between exposed and non-exposed subjects. Considering the design effect (stratified cluster sample) of the study’s complex sample equal to 2.0, the minimum estimated sample size was 672 pupils. We maintained the same proportionality of students aged 12 to 15 years as was observed in the population, using estimates from the Brazilian Institute of Geography and Statistics as a reference point [16]. To account for the possibility of faulty data, losses, or the need for stratifications, we added 30% to this value, for a total minimum sample of 900 adolescents.

Cluster sampling was conducted in two stages. First, we selected the schools (primary sample units) by using the lists of schools available from the National Institute for Educational Studies and Research/Ministry of Education and the Municipal Education Department of São Luís, MA as reference bases. Second, we selected the students (secondary sample units) by using the list of students aged 12 to 15 years enrolled in public and private school networks available at each school selected as a reference bases. The number of students from each school who were enrolled in the study was proportional to the size of the school.

We estimated diagnostic reproducibility (intra- and inter-examiner) by using the Kappa test and the intraclass correlation test, accepting minimum values of 0.7. Two previously trained teams collected the data. Each team consisted of an interviewer/note taker and an examiner (orthodontist), who used a questionnaire and an orthodontic exam sheet, respectively. The questionnaire was used to collect demographic data (e.g., age and sex), socioeconomic data (e.g., administrative category of the school [public or private], educational level of the head of the family (parent or guardian who earns the highest income), household income, history of failing school (students who repeated the same school year one or more times), criteria for economic classification [17], and self-reported skin color [18], -this questionnaire was filled by parents of adolescents- and behavioral data (e.g., adolescent’s perceived need for orthodontic treatment, which was filled in by adolescents).

The criteria for economic classification categorized the economic classes in A-B (class with greater purchasing power), C (class with medium purchasing power) or D-E (lower purchasing power), according the Brazilian Association of Research Companies- ABEP [17]. These criteria consider the possession of consumer goods (television, refrigerator, radio, automobile, maid, washing machine, DVD player and freezer).

Information on oral health-related QOL was obtained via the Portuguese version of the OHIP-14 [12] and was filled by adolescents. The orthodontic exam sheet was used to collect oral health-related data during the clinical exam, such as the occlusal condition according to Angle’s classification [19], the Dental Aesthetic Index (DAI) [20], and the presence of morphological deviations not identified by the previous indicators (e.g., posterior crossbite, posterior open bite, and deep overbite).

### Collection of Oral Health-Related Data

#### OHIP-14

The OHIP-14—Brazilian version is a validated questionnaire about the impact of oral health conditions on QOL [12]. We used this instrument to verify the lived experiences of adolescents in the 12 months before the oral exam. Each question was assigned a weight. The response was calculated on a scale in which the selected code was multiplied by the respective weight of the question. The overall range of the scale was 0 to 28, with index values between 0 and 9 indicating no impact and values between 10 and 28 indicating an impact of oral health on QOL. The higher the value of the index was, the greater the negative impact that oral health had on QOL.

#### Angle’s classification

Molar relationships were evaluated by Angle’s classification as Class I, Class II division 1, Class II division 2, and Class III [21]. For the purposes of analysis, this variable was later categorized as normal, Class I, Class II, and Class III.

#### DAI

The DAI was used to evaluate the presence and seriousness of malocclusion and the normative need for orthodontic treatment. To apply the DAI exams, we used an orthodontic thread (1 mm in diameter and 6 cm in length) and an adapted silicon cursor. We made measurements using an endodontic millimeter ruler [22].

Adolescents with dental aesthetics indices of grade 1 (DAI < 25; no need for treatment) or 2 (DAI = 26–30; elective need) were considered not to have a normative need for treatment. Those with indices of grade 3 (DAI = 31–35; highly desirable need) or 4 (DAI ≥ 36; required need) were considered to have a normative need for treatment.

#### Morphological deviations not identified by above indicators

The DAI only covers changes to teeth in areas anterior to the maxilla and jaw. Therefore, the clinical exam involved a more complete evaluation, to observe the presence of other morphological deviations, such as posterior crossbite, posterior open bite, and deep overbite. Posterior crossbite was defined by the presence of at least one crossed tooth in the premolar or molar area. Posterior open bite was defined by the presence of a space greater than 2 mm between the premolars or the upper and lower molars. Deep overbite was defined when the upper incisors reached the cervical third of the lower incisors, with a trespass greater than 4 mm [23].

### Statistical Analysis

To process the collected variables, we built a database using an Excel spreadsheet and exported the data for analysis by using the Stata for Windows software package (version 11.0). We performed a descriptive analysis of the data, estimating the means, standard deviations, medians, and interquartile ranges, as well as absolute and relative frequencies, with their respective 95% confidence intervals (95% CIs). Differences in the frequency distributions of the qualitative (categorical) variables were analyzed by the Chi-square test.

**Fig 1:**
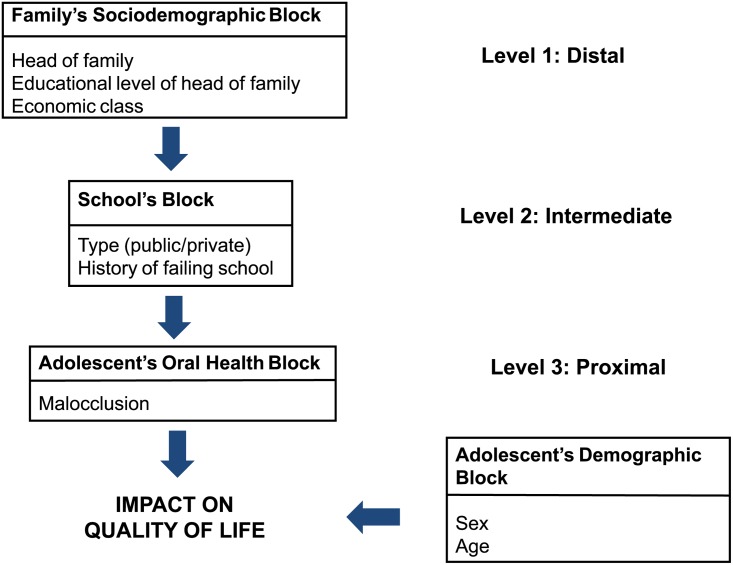
Theoretical model.

We estimated the association of study variables with QOL by the prevalence ratio (PR) and the corresponding interval estimates (95% CIs) by Poisson regression analysis. For adjusted analyses, we used hierarchized modeling (Fig 1). In the adjusted model, variables with p < 0.10 were retained in each block, even when this value was changed by the inclusion of variables from subsequent blocks. An alpha level of 5% was adopted as the criterion for statistical significance. In all analyses, we considered the complex sample design effect (stratified and cluster sample) using the svy commands.

## Results

**Fig 2:**
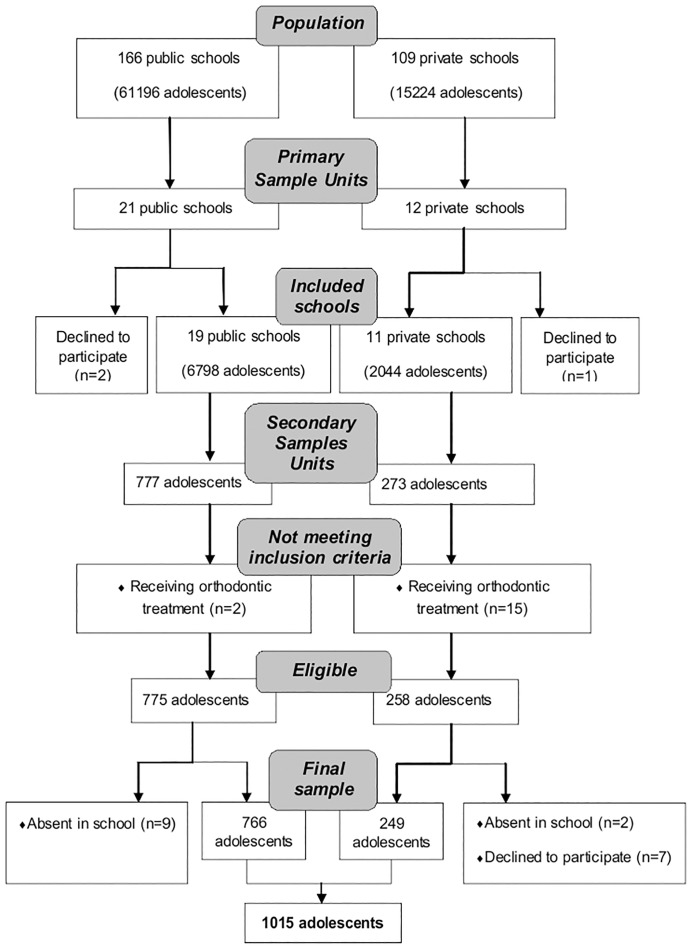
Sample Flow Diagram. São Luís, Brazil, 2012/2013. Adapted from CONSORT 2010.

A total of 1050 adolescents, all 12 to 15 years old, were evaluated for their eligibility to participate in the study; 17 were already receiving orthodontic treatment and were therefore excluded. Of the 1033 eligible adolescents, 7 adolescents refused to participate in the study and 11 adolescents were not present in school at the second evaluation (Fig 2).

The study population comprised 1015 schoolchildren enrolled in 30 schools (19 public and 11 private), 75.4% were from public schools and 68% of economic classes C-D-E. This means that the most of the participants are in a lower socioeconomic condition. Besides, the state in which the study was conducted (Maranhão), is one of the poorest states in Brazil.

The Unified Health System (SUS) does not provide orthodontic treatment for the population in most of Brazilian municipalities. Just recently the Ministry of Health incorporated orthodontic treatment in the list of the services offered in Dental Specialties Centers (CEO) (Ministerial Decree 718-SAS 20/12/2010). However, according to the external evaluation of Access and Quality of Specialized Dental Centers Improvement Program (PMAQ-CEO) [24], the service is still not available in Maranhão, and is offered only in 10% of CEO’s from all over Brazil. Moreover, the few treatments offered do not meet the patient’s demand. Thus, data can be generalized for Brazil and applied to countries with similar socioeconomic situation that do not offer free orthodontic services. Another factor is that most orthodontic treatments start when the person already has more than 18 years old that can work and pay their own orthodontic treatment.

We investigated the association between the variables (sociodemographic and malocclusion) and the normative need for orthodontic treatment by DAI, self-perceived malocclusion and impact of oral health on the QOL of the adolescents (Table 1).

**Table 1:** Variables associated with the normative need for OT by DAI, self-perceived malocclusion and impact of oral health on QOL. Data were obtained from adolescents enrolled in primary education schools in São Luís, Brazil in the 2012/2013 school year.

Variable	Categories (n)	Normative Need for OT by DAI	P	Self-perceived MO	P	Impact on QOL	P
		Yes (%)		Yes (%)		Yes (%)	
**Age (years)**	12 (225)	48.7	0.399	56.6	0.362	13.8	0.046*
	13 (317)	46.4		63.4		14.8	
	14 (226)	43.4		57.5		20.3	
	15 (247)	40.5		62.7		21.1	
**Skin color**	White (249)	49.6	0.189	56.0	0.363	16.5	0.791
	Mixed (623)	43.8		61.6		17.5	
	Black (143)	40.6		62.9		18.2	
**Sex**	Male (503)	48.1	0.021*	56.5	0.022*	14.3	0.022*
	Female (512)	41.5		64.3		20.3	
**Economic class**	A-B (324)	50.5	0.083	57.5	0.276	10.1	0.009*
	C (583)	40.6		62.8		19.7	
	D-E (108)	50.0		56.5		25.9	
**Type of school**	Public (766)	41.2	0.025*	60.3	0.914	20.4	0.010*
	Private (249)	55.6		60.8		8.0	
**Educational level of head of family**	Didn’t complete primary school (146)	45.2	0.180	59.6	0.787	23.3	0.006*
	Completed primary school (245)	43.3		60.4		18.0	
	Completed middle school (431)	42.0		61.9		19.5	
	Completed high school (193)	52.6		57.7		7.2	
**Self-perceived need for OT**	No (402)	39.0	0.003*	—		8.7	<0.001*
	Yes (613)	48.5		—		23.0	
**Normative need for OT by DAI**	No (561)	—		39.0	0.003*	16.0	0.112
	Yes (454)	—		48.5		18.9	
**Malocclusion—Angle’s classification**	Normal (54)	1.8	<0.001*	40.7	0.064	16.7	0.05
	Class I (571)	39.6		60.8		14.7	
	Class II (335)	63.6		62.4		21.5	
	Class III (55)	25.4		63.4		20.0	
**Malocclusion—DAI**	Normal or slight malocclusion (348)	—		51.7	0.002*	16.7	0.641
	Defined malocclusion (213)	—		63.8		15.0	
	Severe malocclusion (199)	—		64.8		19.1	
	Deforming malocclusion (255)	—		66.0		17.8	
**Posterior crossbite**^1^	No (897)	44.4	0.575	59.3	0.296	17.5	0.73
	Yes (118)	47.5		66.1		16.1	
**Posterior open bite**^1^	No (933)	45.5	0.46	60.7	0.516	16.7	0.091
	Yes (82)	35.4		57.3		24.4	
**Overbite**^1^	No (692)	36.8	<0.001*	60.3	0.760	18.4	0.241
	Yes (318)	61.6		61.3		14.8	

OT = orthodontic treatment. MO = malocclusion.

*Statistically significant difference (p < 0.05).

^1^Variables not identified by the DAI.

Adolescent girls reported more malocclusions than adolescent boys (64.3% versus 56.5%, P = 0.022), however, they had less normative need for orthodontic treatment (41.5% versus 48.1%, P = 0.021). Students of private schools had higher normative need for orthodontic treatment (55.6% versus 41.2%, P = 0.025), but the perception of orthodontic problems was similar between the two types of school (P = 0.914). There was an association between the need for orthodontic treatment according to DAI and the self-perceived need (P = 0.003). The malocclusion by Angle criterion was associated with malocclusion according to DAI (P <0.001) but not with the self-reported malocclusion (P = 0.064).

Age (p = 0.046), sex (p = 0.022), economic class (p = 0.009), type of school (p = 0.010), educational level of the head of the family (p = 0.006), and self-perceived need for orthodontic treatment (p < 0.001) showed significant differences in the impact on QOL in the frequency distributions between the groups.

Poisson regression analysis was used to compare the sociodemographic variables and the impact on QOL (Table 2). Sex (PR = 1.40; 95% CI = 1.05–1.89), head of the family (PR = 1.52; 95% CI = 1.03–2.23), and educational level of the head of the family (PR = 0.32; 95% CI = 0.17–0.61) were associated with an impact of oral health on QOL.

**Table 2:** Poisson regression analysis between sociodemographic variables and the impact of oral health on QOL. Data were obtained from adolescents enrolled in primary education schools in São Luís, Brazil in the 2012/2013 school year.

Variable	Association between sociodemographic variables and impact on QOL			
	Unadjusted		Adjusted^1^	
	PR	95% CI	PR	95% CI
**Sex**				
Male	1.00		1.00	
Female	1.42	1.05–1.91*	1.40	1.05–1.89*
**Head of the family**				
Father	1.00		1.00	
Mother	1.28	0.86–1.90	1.19	0.83–1.72
Other	1.66	1.08–2.54*	1.52	1.03–2.23*
**Educational level of head of family**				
Did not complete primary school	1.00		1.00	
Completed primary school	0.77	0.47–1.26	0.78	0.47–1.29
Completed middle school	0.84	0.53–1.32	0.86	0.54–1.39
Completed high school	0.31	0.16–0.59*	0.32	0.17–0.61*

OT = orthodontic treatment.

*Statistically significant difference (p < 0.05). Only the variables that remained in adjusted model are presented.

^1^Adjusted for the adolescent’s sex, head of the family, and educational level of the head of the family.

Table 3 shows the measurement of the association obtained by the Poisson regression analysis between the occlusal variables and the impact on QOL. A normative need (PR = 1.27; 95% CI = 1.03–1.56) and a self-perceived need (PR = 2.54; 95% CI = 1.81–3.56) for orthodontic treatment were associated with an impact on QOL. The other occlusal variables evaluated did not show significant associations.

**Table 3:** Poisson regression analysis between normative/self-perceived need for treatment of malocclusion and the impact of oral health on QOL. Data were obtained from adolescents enrolled in primary education schools in São Luís, Brazil in the 2012/2013 school year.

Variable (Reference category)	Comparing category	Association between malocclusion variables and impact on QOL			
		Unadjusted		Adjusted^1^	
		PR	95% CI	PR	95% CI
**Angle (Normal)**	Class I	0.88	0.53–1.46	0.90	0.52–1.53
	Class II	1.29	0.75–2.21	1.24	0.71–2.16
	Class III	1.20	0.56–2.55	1.17	0.52–2.61
**Loss of teeth (No)**	Yes	1.50	0.97–2.33	1.35	0.89–2.05
**Anterior crowding (No)**	Yes	1.04	0.83–1.30	1.15	0.94–1.41
**Anterior spacing (No)**	Yes	0.81	0.59–1.10	0.84	0.60–1.17
**Incisal diastema (No)**	Yes	0.79	0.56–1.13	0.81	0.56–1.16
**Maxillary misalignment (No)**	Yes	1.11	0.91–1.35	1.14	0.93–1.39
**Misaligned jaw (No)**	Yes	1.05	0.79–1.39	1.15	0.88–1.50
**Maxillary overjet (No)**	Yes	0.75	0.53–1.08	0.85	0.59–1.22
**Mandibular overjet (No)**	Yes	1.59	0.94–2.70	1.46	0.89–2.38
**Anterior open bite (No)**	Yes	1.25	0.72–2.18	1.20	0.67–2.13
**AP molar relationship (Normal)**	Half cuspid	1.19	0.78–1.79	1.06	0.70–1.59
	One cuspid	1.24	0.78–1.97	1.15	0.70–1.90
**Posterior crossbite (No)**	Yes	0.92	0.56–1.51	0.95	0.58–1.56
**Posterior open bite (No)**	Yes	1.46	0.94–2.25	1.35	0.88–2.06
**Overbite (No)**	Yes	0.80	0.54–1.17	0.97	0.68–1.39
**Normative need for OT by DAI (no MO to slight MO)**	Defined MO	0.90	0.58–1.39	0.94	0.62–1.42
	Severe MO	1.14	0.79–1.65	1.22	0.85–1.75
	Deforming MO	1.18	0.96–1.45	1.27	1.03–1.56*
**Self-perceived need for OT (No)**	Yes	2.64	1.90–3.68*	2.54	1.81–3.56*

MO = malocclusion, OT = orthodontic treatment, AP = antero-posterior.

*Statistically significant difference (p < 0.05).

^1^Adjusted for the adolescent’s sex, head of the family, and educational level of the head of the family.

## Discussion

Adolescence is a period of transformation, characterized by an emotional reorganization involving various internal and external conflicts. Because of these factors, there has been increasing interest in the impact of malocclusion on the adolescent’s psychosocial well-being [25]. Therefore, we not only evaluated the impact of malocclusion, but also the impact of the perceived need for orthodontic treatment, on the adolescents’ QOL. When classified according to Angle’s classification, malocclusion did not affect the adolescents’ QOL. However, when evaluated by focusing on dental appearance using the DAI, malocclusion had a negative impact on the adolescents’ QOL.

These results differ from those of some other studies [3,7,26,27], which did not find an association between the normative need for orthodontic treatment and the impact on QOL. One possible explanation for this difference might be the fact that the OHIP-14 questionnaire was not developed to measure the impact of orthodontic problems on QOL specifically. This instrument measures the impact of oral health on QOL in a general way, capturing effects attributed to malocclusion and dental appearance, as well as other oral conditions, including pain, phonation problems, periodontal disease, and caries. [28–30].

We found that 44.72% (n = 454) of the adolescents had a normative need, but 60.39% (n = 613) of adolescents had a self-perceived need, for orthodontic treatment. This result differs from studies showing that normative clinical criteria overestimate problems compared to a person’s self-perception [31,32]. Even if a physical problem affects a person’s subjective perception of well-being, the impact on QOL also depends on their expectations and preferences, their material, social, and psychological resources, and, above all, their social and cultural values [28–30].

There are many reasons why an adolescent may feel a need for orthodontic treatment and, in many cases, these reasons are not related to the seriousness of malocclusion [33]. We found that the self-perceived need for orthodontic treatment was associated with the impact on QOL, consistent with the findings of other investigations [3,7,26,31,34]. Adolescents’ self-perceived need for orthodontic treatment appeared to be associated with aesthetic factors. Specifically, the impact on QOL was associated with malocclusion when measured via the DAI, but not when measured via Angle’s classification. Marques et al. (2006) [3] obtained similar results, also finding that adolescents attribute importance to the appearance of their teeth [26,35], which, in turn, is influenced by their dissatisfaction with their appearance and manifests itself in the desire for orthodontic treatment [3,36,37].

When the variables for malocclusion that comprise the DAI were analyzed separately, we did not observe an association between these variables and the impact on QOL. Our findings differ from those of previous studies that found an impact on QOL to be associated with misalignment of the maxillary teeth [3] or loss of the anterior teeth [38, 39]. The differences between the findings may be partly explained by the different forms of malocclusions’ classification. The Vargas and Paixão’s study [38] used the rating of the self-reported MO, while in others studies [3, 39] participants were examined using different indicators (DAI and OIDP). In our evaluation, we found a relation between MO and QoL using both classifications: DAI and self-reported MO. However, DAI was just associated to QoL in deforming category (in a mandatory orthodontic treatment status). We believe that only a specific problem of MO is not enough to impact on QOL. Nevertheless, when the aesthetic commitment is greater (affecting different areas of DAI), the impact becomes noticeable, as noted in our results.

The relation between MO and QOL was higher when the MO was classified according to self-reported criteria. Thus, we suggest that the negative impact of a self-perceived need for orthodontic treatment on QOL does not necessarily correspond to the orthodontist’s assessment of the malocclusion [33]. Other factors, such as the adolescent’s desire to be in a high socioeconomic position or to be fashionable [40,41], are important, given that socioeconomic conditions had a negative impact on QOL.

Our study showed an association between socioeconomic conditions and an impact on QOL through the variables of the head of the family and the educational level of the head of the family. Although some authors [7,42] have claimed that a person’s socioeconomic condition does not negatively affect QOL, our results corroborate those of Davey-Smith, Blane, and Bartley (1994) [43], Piovesan et al. (2010) [44], Scapini et al. (2013) [8], Benson et al. (2014) [45], and Tuchtenhagen et al. (2015) [25]. They showed that a low level of education on the part of the head of the family directly and negatively affects the formulation of concepts of self-care in health. Adolescents from such families are more susceptible to oral conditions that can negatively influence QOL, such as poorer oral health; in other words, they have a greater experience of caries and periodontal disease [8,26,43–45]. In addition, some authors have noted that socioeconomic conditions are directly related to the presence of harmful oral habits, which could influence the development of malocclusion [46].

The variables of skin color and age did not show an association with the QOL related to the adolescents’ oral health, similar to the studies by Feu et al. (2010) and Scapini et al. (2013) [7,8]. The variable of sex showed an association with an impact on QOL. Girls said that oral health had a higher impact on QOL than boys, similar to the study by Ukra et al. (2013) [9]. The need for orthodontic treatment and its impact on QOL seemed to be associated with cosmetic factors. Thus, these results suggest that a person’s sex influences the QOL of adolescents who are preoccupied with their appearance and who desire orthodontic treatment, as other studies have also reported [7,47,48].

The clinical implications of this study were that quality of life is more associated with aesthetic and socioeconomic condition than with the functional problems of malocclusion. The study’s cross-sectional design could be considered the limitation of this research, although it is not an important one because occlusion is established by the age range examined in this study and longitudinal tracking would not change the results significantly.

## Conclusion

Malocclusion, represented by the normative need and the self-perceived need for orthodontic treatment, has a negative impact on the QOL of adolescents. This impact is mostly associated with aesthetic changes related to malocclusion and to the adolescent’s socioeconomic condition. There was no relation between QOL and malocclusion when the latter was measured by Angle classification.
